# Worldwide Variations in Fluoride Content in Beverages for Infants

**DOI:** 10.3390/children10121896

**Published:** 2023-12-07

**Authors:** Eleonor Velez-León, Edisson-Mauricio Pacheco-Quito, Mario Díaz-Dosque, Daniela Tobar-Almache

**Affiliations:** 1 Unidad Académica de Salud y Bienestar, Facultad de Odontología, Universidad Católica de Cuenca, Cuenca 010105, Ecuador; mvelezl@ucacue.edu.ec; 2 Grupo de Investigación Innovación y Desarrollo Farmacéutico en Odontología, Facultad de Odontología, Jefatura de Investigación e Innovación, Universidad Católica de Cuenca, Cuenca 010105, Ecuador; 3 Latin American Network of Research on Fluorides and Dental Fluorosis, Cartagena 130009, Colombia; mrdiaz@uchile.cl (M.D.-D.); dtobara@odontologia.uchile.cl (D.T.-A.); 4 Laboratory of Pharmacology, Institute for Research in Dental Sciences (ICOD), Faculty of Dentistry, University of Chile, Olivos 943, Independencia, Santiago 8380544, Chile

**Keywords:** milk, formula milk, fluoride, fluorosis

## Abstract

In situations where breastfeeding is impractical, milk formulas have emerged as the primary choice for infant nutrition. Numerous global studies have scrutinized the fluoride content in these formulas, uncovering fluctuations in fluoride levels directly associated with the method of preparation. This variability poses a potential risk of elevated fluoride concentrations and, consequently, an increased susceptibility to dental fluorosis in infants. The primary objective of this review is to intricately delineate the fluoride content in dairy formulas and emphasize the variability of these values concerning their reconstitution process. The review’s findings reveal that, among the 17 studies assessing fluoride levels in infant formula, milk-based formulas exhibit a range of 0.01–0.92 ppm, with only two studies exceeding 1.30 ppm. Conversely, soy-based formulas demonstrate values ranging from 0.13–1.11 ppm. In conclusion, the observed variability in fluoride levels in infant formulas is ascribed to the choice of the water source employed in the preparation process. This underscores the paramount importance of meticulously adhering to recommendations and guidelines provided by healthcare professionals concerning the utilization of these formulas and their meticulous reconstitution.

## 1. Introduction

Fluorine (F) is a chemical element that belongs to the halogen family and is found in the form of a gas. Fluoride is derived from fluorine, a compound naturally abundant in the Earth’s crust and present in various sources, such as groundwater, beverages, and food [1,2]. Fluoride possesses beneficial properties for preventing tooth decay. Nevertheless, excessive consumption during the formative years of tooth development can have adverse effects on human health and lead to dental fluorosis [3,4]. Hence, while fluoride plays a substantial role in dental remineralization, it concurrently presents certain drawbacks that warrant careful scrutiny. For instance, the consumption of appropriate levels of fluoride, between 0.5 and 1.0 ppm, through drinking water has been proven to be beneficial for the prevention of dental caries. However, excessive consumption has been shown to cause dental fluorosis [5,6]. Consequently, finding an appropriate balance in fluoride intake is essential to take advantage of its beneficial effects without producing toxicity [7].

Many studies have demonstrated that appropriate levels of fluoride from various sources have a positive impact on reducing dental caries [8,9]. These benefits are attributed to various mechanisms, including the inhibition of bacterial plaque formation, the subsequent prevention of demineralization, and the enhancement of remineralization [10,11,12]. Moreover, there is evidence supporting the importance of exposing individuals to optimal fluoride levels during infancy and early childhood, as this will contribute to proper dental development and a lower incidence of dental caries in adulthood [13].

Systemic fluoride, one of the methods of fluoride use, has traditionally been employed for cavity prevention and can be obtained from various sources, including fluoridated water and salt, milk, dietary supplements, and processed foods, juices, and beverages prepared with fluoridated water [3,8,14,15]. For children in their early years, all of these sources of fluoride should be monitored to prevent possible complications, taking into account both the concentration and frequency of exposure [10].

A child’s primary source of nutrition in the early stages of life is breast milk, which contains 0.005–0.01 mg/L of fluoride [16]. This is the most effective means of safeguarding the health of both mothers and infants, fostering their robust growth and development. In a 2018 analysis, it was emphasized that approximately 7.6 million children worldwide are deprived of the advantages of breastfeeding each year. This highlights a significant issue in child healthcare and nutrition [17]. A study conducted by UNICEF in 2017, which examined breastfeeding practices in 194 countries, has revealed alarming statistics. Only 40% of children under six months are exclusively breastfed, and only 23 countries reported exclusive breastfeeding rates exceeding 60%. This can be attributed to various factors, including the lack of appropriate policies to support breastfeeding for approximately 830 million working women worldwide [18]. Additionally, the use of post-pregnancy medications, women returning to work, and the presence of HIV are notable factors contributing to this situation [19].

Consequently, milk-based formulas have become the best option for infant feeding when they are not being breastfed. Worldwide, a plethora of studies has investigated the fluoride concentration in milk-based formula. These studies have demonstrated that fluoride levels can vary, depending on the reconstitution method, potentially leading to increased fluoride concentrations [20]. The objective of this review is to assess the fluoride content in infant formulas and examine the extent to which these values fluctuate based on the reconstitution process, potentially leading to a risk of dental fluorosis.

### 1.1. Dental Fluorosis: Risk Factors

The administration of fluoride is effective in preventing dental caries when administered in appropriate amounts and when following the guidance of health professionals. Otherwise, it could lead to fluorosis [21,22,23,24].

Dental fluorosis is a condition that can arise due to excessive fluoride exposure during tooth development, typically occurring in childhood. The severity of this condition can vary depending on the quantity of fluoride and the duration of exposure. At a molecular level, excessive systemic fluoride consumption exerts adverse effects on ameloblast development. It can lead to DNA damage, apoptotic cell death, endoplasmic reticulum stress, and mitochondrial dysfunction, in this way compromising ameloblast function [25]. This disruption subsequently interferes with the typical synthesis and secretion of enamel-associated proteins due to impaired endoplasmic reticulum function [25]. The clinical manifestations may include the development of horizontal white lines on the tooth’s surface or the formation of white, chalk-like spots. In certain instances, the entire tooth’s surface may appear chalky white, with a loss of its original translucency. In more severe cases, staining ranging from yellow to brown may become apparent on the tooth’s enamel [26].

The prevalence of dental fluorosis has increased worldwide due to increasing levels of fluoride exposure. In countries such as Ecuador [27], Tanzania [28], Thailand [29], Mexico [30], China [31], the United States [32], and Chile [33], prevalence rates of 63.7%, 89.7%, 53.4%, 30.5%, 35.6%, and 53.31% have been reported among children aged 6 to 12 years, respectively. This increase has predominantly affected milder forms of fluorosis in communities exposed to fluoride. It is important to emphasize that this does not pose a significant health risk to individuals. Instead, it underscores the critical need for strict control measures in water, salt, and milk fluoridation programs, especially in regions where the use of drinking water as a fluoride carrier is not possible.

Several studies conducted in various regions around the world have reported cases of dental fluorosis in cities whose populations consume drinking water with fluoride concentrations lower than 1.0 mg/L [34,35]. Consequently, the Department of Health and Human Services in the United States and New Zealand recommends fluoride concentrations of 0.7 mg/L in drinking water, in line with other countries like Chile, which sets optimal fluoride concentration values for drinking water ranging from 0.6 to 1.0 mg/L [36,37,38]. These findings suggest that the optimal fluoride dosage level in drinking water may vary due to various factors, including local climate conditions and food cooking and processing methods [39].

Breast milk, with minimal fluoride content, is the primary source of nourishment for infants and toddlers. In cases where it is unavailable or insufficient, infant formulas become necessary alternatives [34,35].

### 1.2. Recommended Levels of Fluoride for Infants and Children: International Regulations

Initiating food and nutrient intake plays a critical role in promoting overall health and preventing diseases [40]. A notable example can be found in formulations tailored for the pediatric market, whether in powdered, standard liquid, or ready-to-feed liquid forms. These formulations are of particular significance in terms of their contribution to fluoride intake. Scientific research has revealed that certain brands contain exceptionally high levels of fluoride. Consequently, when combined with optimally fluoridated water, they result in fluoride concentrations that exceed the recommended levels within the formula composition [41,42,43].

Institutes and health associations have defined intake levels applicable to all age groups. These recommended thresholds are based on estimates that have demonstrated their effectiveness in decreasing the incidence of dental caries [44,45,46].

Several organizations from various countries have issued guidelines regarding the use of infant formula and the potential risk of enamel fluorosis [47].

In accordance with the directives of the Commission of the European Communities, regulations stipulate that the fluoride content in infant formulas and follow-on formulas should not exceed 100 µg/100 kcal when prepared according to the manufacturer’s instructions [48]. Nevertheless, both the European Society of Pediatric Gastroenterology, Hepatology and Nutrition and the Federation of International Societies of Pediatric Gastroenterology, Hepatology, and Nutrition have expressed concerns regarding high fluoride intake during early childhood, which elevates the risk of dental fluorosis [49]. Consequently, it is strongly advised that the maximum allowable fluoride levels in infant formulas is minimized, staying below 60 μg/100 kcal [49].

In the United States, the American Dental Association has published a provisional guideline regarding fluoride intake for infants and children. This guideline suggests that in areas with optimal levels of fluoride in tap water (0.7 ppm), which has been demonstrated to be beneficial in reducing cavities, it is safe for infants and children from 6 months to 6 years of age to ingest 0.12 mg of fluoride per kg of body weight per day [50].

The European Food Safety Authority (EFSA) [51] established the maximum tolerable level of daily fluoride intake (F) as 0.1 mg of F per kg of body weight for children aged 1 to 8 years [34]. On the other hand, the United States Environmental Protection Agency (EPA) has established a No Observed Adverse Effect Level (NOAEL) of 0.06 mg of F per kg of body weight per day [52].

In the case of South America, limited information is available regarding the recommended fluoride doses in pediatric formulas, as studies are scarce in the nutritional and dental fields that establish a connection between dental fluorosis and fluoride intake. In the case of Chile, according to the Standard for the Use of Fluoride in Dental Prevention provided by the Ministry of Public Health, fluoride concentrations in infant milk range between 3.15 and 4.25 mg/L. Consequently, the fluoride amount provided per commonly consumed portion (200 mL) ranges from 0.63 to 0.85 mg [53].

### 1.3. Fluoridated Milk

Fluoridated milk is an additional food that has been endorsed by the World Health Organization (WHO) for use in infant feeding [54]. Fluoridated milk is distributed in 200 mL containers containing 5 ppm of fluoride, which corresponds to 1 mg of fluoride in the container [55].

The utilization of milk as a means to introduce fluoride into dental public health initiatives is highly compelling. To begin with, milk fluoridation has proven to be a thousandfold more cost-effective than water fluoridation, as demonstrated by the outcomes of a cost comparison study. This is particularly significant for developing nations like Chile [56].

Furthermore, milk holds a pivotal place in the diets of children and has served as a nutritional supplement for numerous populations, especially those in vulnerable circumstances, over many years. The advantages of milk consumption encompass a range of benefits, notably the promotion of bone health through mineral acquisition and the development of increased muscle mass [57].

Fluoridated milk is available in various formats, which include liquid forms (pasteurized, ultra-high-temperature pasteurized, and sterilized) and powdered varieties. The primary method for milk fluoridation typically entails the incorporation of sodium fluoride, except in Chile, where disodium monofluorophosphate is utilized. The choice of fluoridation method is influenced by various factors, such as the composition of dietary supplements, resource availability, and the necessary fluoride concentration, which is determined by the age of children, among other considerations [58].

## 2. Materials and Methods

This research was conducted through a comprehensive literature search in accredited databases, including PubMed, Scopus, ScienceDirect, and Web of Science, using MeSH terms.

To ensure the quality and relevance of the selected articles, inclusion criteria were established based on keywords such as “fluoride”, “infant formulas”, or “milk”, excluding “humans”. During the initial review, two reviewers assessed all abstracts. Subsequently, the research group conducted a full review of the articles to select those that met the inclusion and exclusion criteria. If there were discrepancies between two reviewers regarding the data or results of individual articles, a conflict resolution process was implemented. This included an in-depth discussion of the discrepancies, allowing each reviewer to present arguments and evidence to support her position. If differences persisted, a third reviewer could be consulted to evaluate the article in question and make a final decision. Relevant information covering the period from 2000 to 2022 was collected.

The exclusion criteria were based on the following aspects: studies that were not relevant to the objectives of the review, articles that were not available in the mentioned databases, gray literature, studies published outside the search period, incomplete research (articles that lacked complete data), and those that belonged to specific categories of studies, such as literature reviews, systematic reviews, meta-analyses, letters to the editor, conference abstracts, or trial registries that had not undergone peer review. The inclusion criteria encompassed studies that investigated the association between fluoride and milk-based formulas while providing data regarding fluoride concentrations in these milk-based formulas. It is noteworthy that no articles were excluded based on their language of publication; we included articles in both Spanish and English. Furthermore, we did not exclude any studies based on their research design.

**Figure 1:**
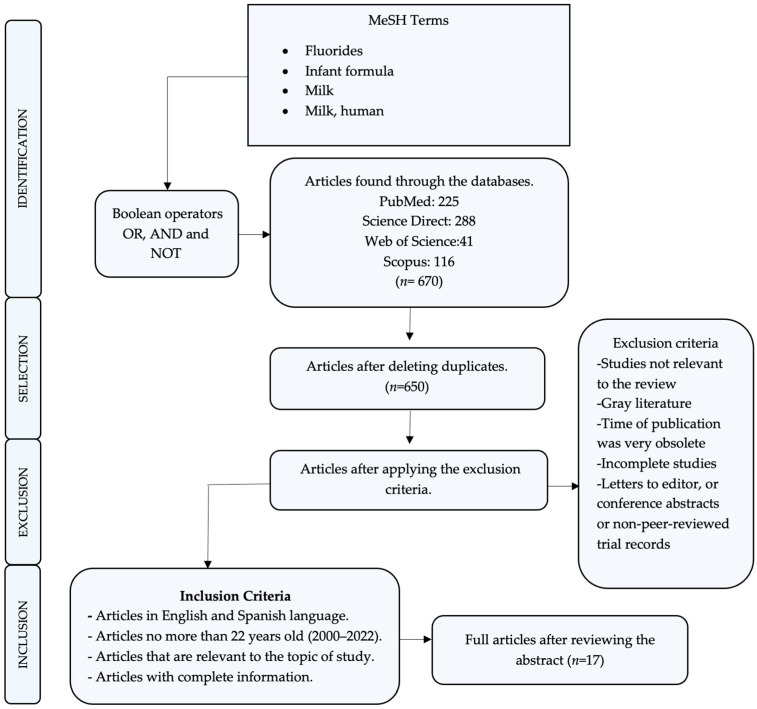
Search flowchart.

Based on the described methodology and the scientific evidence, Figure 1 represents the PRISMA flow diagram, with the results obtained in this review.

## 3. Results

### 3.1. Identification and Selection of Studies

In the initial phase of the investigation, researchers utilized digital search engines to identify a total of 650 studies from reputable databases, including PubMed, Scopus, ScienceDirect, and Web of Science. Employing a rigorous screening process based on title and abstract, 600 studies were excluded. Subsequently, a meticulous examination of the complete documents from the remaining 50 publications was conducted, resulting in the exclusion of 33 studies due to their failure to meet predetermined methodological standards. Bibliographic augmentation was deemed unnecessary, as additional articles lacked relevance to the study’s focus or were already incorporated. Ultimately, 17 studies, all published between 2000 and 2022, were included in this systematic review. The primary focus of these studies was the determination of fluoride concentration in infant formulas. Each contributing author reported explicit values regarding fluoride concentration in the scrutinized infant formulas. However, a subset of five authors [59,60,61,62,63] executed reconstitutions of these formulas using water, thereby introducing variability in the resulting fluoride levels.

### 3.2. Fluoride in Infant Formulas

A summary of studies that have investigated fluoride concentrations in milk-based formulas in different regions of the world is presented in Table 1.

In the investigation conducted by Koparal et al. [64] in the year 2000, which focused on examining the fluoride content in various infant food sources, including milk-based formulas, it was observed that infants who were fed with milk-based formulas could potentially experience higher fluoride intake, ranging from 0.021 to 0.118 ppm, compared to breastfed infants, who exhibited a level of 0.022 ppm.

Similarly, Clifford et al. [59] sought to determine the fluoride content in both milk- and soy-based powdered formulas. Their findings revealed that when the milk-based formula was reconstituted in fluoride-free water, it averaged 7.09 μg F/100 mL. In contrast, the soy-based powdered formula exhibited an average fluoride content of 1.0 mg/L. This resulted in a mean daily fluoride intake from infant formulas, in milligrams of fluoride per average weight for age, ranging between 0.04 and 1.04 mg F/day for dairy and milk-based formulas and from 0.11 to 1.12 mg F/day for soy-based infant formulas, indicating minimal fluoride intake for infants consuming milk-based formulas compared to those consuming soy-based powdered formulas. When both milk and soy powdered formulas were reconstituted with water containing 0.7 mg/L fluoride, intake estimates exceeded recommendations but stayed below the upper limit for age. In the case of reconstitution with water containing 1 mg/L fluoride, estimates surpassed both the recommendations and the upper limit for all age groups, suggesting a slight risk of dental fluorosis.

In 2016, Bussell et al. [20] conducted a study in the United Kingdom, focusing on fluoride concentrations in milk-based powders. The investigation indicated that commercial formulas reconstituted with 0.02 ppm of fluoridated water and ready-to-drink milk exhibited low levels of fluoride. The authors estimated that powdered milk-based formulas contribute to an intake of 0.0034 mg F/kg/day (median) from birth to 6 months of age. These levels were considered unlikely to pose a risk for dental fluorosis development. However, variations in composition, manufacturer, and discrepancies between measured and labeled fluoride concentrations were noted.

Contrasting with the 2009 studies [59], this investigation did not involve the reconstitution of infant formulas with different fluoride levels. However, it emphasized that fluoride intake predominantly relies on the concentration of fluoride in the water used for reconstitution rather than the concentration in the powdered formula itself. The importance of researching diverse populations or areas susceptible to fluoride exposure was highlighted, and a relevant study by Valdez Jiménez et al. [65], exploring fluoride exposure through milk in children residing in an area with endemic fluorosis, was emphasized.

Valdez Jiménez et al. [65] revealed that fluoride levels in infant formulas reconstituted with public water, as well as in pasteurized cow’s milk and raw cow’s milk, exceeded recommended limits, potentially leading to dental fluorosis in consumers. The importance of ensuring that dairy products and other items relying on water for production meet regional quality standards was underscored, especially in regions with potentially contaminated water sources. The study showed variations in fluoride concentration in various types of milk, with breast milk exhibiting a concentration of 0.4 mg F/L and formula milk reconstituted with public water having an average of 0.9 mg F/L.

In 2019, Y. Agha et al. [10] assessed fluoride concentrations in powdered milk-based infant formulas. The study demonstrated that when reconstituted with deionized water, powdered milk-based formulas contained fluoride levels below the recommended daily maximum limit, averaging 0.19 ppm. However, in everyday scenarios, caregivers commonly reconstitute baby formula using tap water or bottled water.

Yanagida et al. [66] analyzed five infant formulas, revealing a mean fluoride content of 0.90 μg/g in milk-based powdered infant formulas when reconstituted in water with a fluoride concentration of 0.0524 μg/mL. The estimated daily intake (EDI) of fluoride from powdered infant formulas ranged between 77.86 and 264.33 μg, depending on the brand. Similar to the study by S. Agha et al. [10], these studies did not address the variability in fluoride levels that may arise due to the type of water used to reconstitute milk-based powdered infant formula.

The most recent study in 2022 by Chandio et al. [67] updated existing knowledge in this field, revealing fluoride content values in milk powder for infant formulas (reconstituted with distilled water) and ready-to-feed formulas (RTF) in the ranges of 0.03 ppm and 0.27 ppm. Soybean RTF averaged higher at 0.56 ppm. This presented a contrast with the outcomes of a prior study conducted in the same country in 2009 [59], where fluoride values exhibited a range of 0.09 to 0.171 ppm.

Soy-based infant formulas, gaining popularity as an alternative to milk-based formulas, demonstrated fluoride concentrations ranging from 0.21 to 0.55 ppm [67,68], suitable for individuals with lactose intolerance or milk allergies. In contrast, ready-to-feed milk-based formulas for infants had fluoride concentrations ranging from 0.03 to 0.15 ppm [20,60]. This variation in fluoride levels was also observed in reconstituted milk-based and soy-based formulas, containing between 0.06 and 0.27 ppm and 0.13 to 0.47 ppm [20,41], respectively, when reconstituted with deionized water. Notably, when soy-based formulas were reconstituted with fluoridated water, fluoride levels experienced a significant increase, reaching 1.11 ppm fluoride, contradicting the results of Clifford’s [59] research, which indicated fluoride levels of 0.17 ppm when reconstituting soy-based formulas.

The way infant formula is prepared significantly influences a child’s fluoride intake, as evidenced by research conducted by Opydo-Szymaczek et al. [61] and Cressey et al. [69]. These studies involved the preparation of infant formulas using water with varying fluoride concentrations, demonstrating that fluoride levels were notably higher when water with fluoride levels between 0.7 and 1.0 mg/L was utilized. Collectively, these findings indicate that the consumption of infant milk reconstituted with fluoridated water exceeds the recommended fluoride levels.

Significant variations in fluoride content exist in the water used to reconstitute infant formulas, as demonstrated by Steinmetz et al. [70]. Their study focused on assessing fluoride levels in bottled water, analyzing 20 different brands. The findings revealed fluoride concentrations ranging from 0.006 to 0.740 mg/mL. Notably, except for one brand, most bottled water brands exhibited fluoride concentrations below 0.7 mg/mL, with 16 brands having concentrations below 0.22 mg/mL. These comparisons suggest that most of the examined bottled waters align with recent recommendations from the American Dental Association (ADA) and the US Food and Drug Administration (FDA) [46,71].

## 4. Discussion

The contribution of fluoride content in the water used to reconstitute infant formulas significantly shapes the overall daily fluoride intake in infants, who primarily depend on these formulas as their main nutritional source [10].

Fluctuations in fluoride concentration in water can be attributed to various factors, notably the influence of the specific environment where the study took place. This environment, characterized by a rural setting and endemicity to hydrofluorosis, has been further impacted by the overexploitation of aquifers supplying water to various Mexican cities. Jiménez et al.’s investigation reveals that this overexploitation has led to the extraction of water from increasingly deeper wells [65]. The ingestion of elevated fluoride concentrations results from the precipitation of fluoride ions in the depths of water sources, significantly impacting fluoride levels in various infant foods, with infant formulas being particularly vulnerable to this phenomenon [72].

The potential impact of infant formula on the systemic intake of fluoride in infants, influenced by variations in fluoride levels based on the water source used for reconstitution, underscores the need for vigilant monitoring to prevent adverse effects, especially during early developmental stages, such as neurotoxicity and dental fluorosis [73].

Dentists and pediatricians play a crucial role in effectively communicating ADA guidelines to their patients. These guidelines emphasize the benefits of breastfeeding, highlighting that the utilization of ready-to-use infant formulas maintains fluoride intake within recommended thresholds for infants. In contrast, powdered formulations offer flexibility in reconstitution with fluoride-free or low-concentration water [74].

In providing guidance to parents and caregivers, healthcare professionals must consider the quantity, duration, and timing of fluoride consumption, recognizing the observed correlation between fluoride ingestion during tooth development and the risk of fluorosis [75].

Accessible resources, exemplified by the public water system report from local water utilities [50], serve as valuable tools for healthcare professionals and parents to understand fluoride concentrations in water. These reports consistently confirm that the fluoride content in most water sources adheres to established upper limits, with occasional deviations predominantly affecting smaller water suppliers [76,77,78].

While the fluoride content in powdered infant formulas and other region-specific presentations generally adheres to required guidelines for fluoride administration, it is crucial to underscore the limited universality of these findings due to the reliance on samples from local producers in previous studies. Furthermore, fluoride concentrations in infant formulas can exhibit variations, even in studies conducted in the same country but in different years [59,67]. This underscores the need for continuous updates, as fluoride concentrations in formulas have the potential to change over time, influenced by factors such as the manufacturer.

As a result, there is a growing demand for research in this field, specifically to assess fluoride concentrations in formula milk consumption, particularly in South America. This research will serve as the cornerstone for developing health strategies and policies, providing precise recommendations for fluoride intake to ensure levels that are both safe and adequate, contributing to enhanced oral health in the pediatric population.

**Table 1:** Summary of Global Studies on Fluoride Concentration in Infant Milk Formula.

Country	Milk Presentation	Number of Samples	F Concentration (ppm)/Mean (SD)	F Concentration (ppm) Median (Range)	Reference
United Arab Emirates	- Milk-based formula powder (NFW) *.	24	0.19 ppm (NR) ^•^	NR ^•^ (0.0–0.4)	[10]
United Kingdom	- Milk-based formula powder (WD) ^-^. - Soy-based powdered (WD) ^-^. - Milk-based formula powder (NFW) *. - Soy-based powdered (NFW) *. - Milk-based formula powder (WF) > (Water with 0.90 ppm F). - Soy-based powdered (WF) > (Water with 0.90 ppm F).	18	0.06 ppm (0.04) 0.13 ppm (0.08) 0.15 ppm (0.07) 0.20 (0.08) 0.91 ppm (0.22) 1.11 ppm (0.17)	0.04 (0.02–0.18) 0.16 (0.00–0.18) 0.14 (0.07–0.32) 0.22 (0.12–0.27) 0.92 (0.49–1.40) 1.11 (0.93–1.28)	[63]
Turkey	- Milk-based formula powder (DW) ^+^.	10	0.06 ppm (NR) ^•^	NR ^•^ (0.11–0.02)	[64]
Japan	- Milk-based formula powder.	5	0.90 ppm (0.21)	NR ^•^ (0.25–1.56)	[66]
Brazil	- Milk-based formula pow-der (WD) ^-^. - Milk-based formula powder (TW) ^1^. - Milk-based formula powder (WD) ^-^. - Soy-based milk (WD) ^-^.	19 10	0.55 ppm (NR) ^•^ 1.3 ppm (NR) ^•^ 0.18 ppm (NR) ^•^ 0.47 ppm (NR) ^•^	NR ^•^ (0.01–1.10) NR ^•^ (0.74–1.85) NR ^•^ (0.04–0.32) NR ^•^ (0.25–0.70)	[79,80]
United Kingdom	- Soy-based milk. (TW) ^1^. - Milk-based formula powder (TW) ^1^. - Ready-to-drink milk.	47	0.12 ppm (NR) ^•^ 0.01 ppm (NR) ^•^ 0.14 ppm (NR) ^•^	NR^•^ (0.00–0.25) 0.01 (0.00–0.03) 0.01 (0.00–0.28)	[20]
Mexico	- Milk-based formula powder (TW) °.	47	0.9 ppm (0.8)	NR ^•^ (0.2–3.9)	[65]
United States	- Milk-based formula powder. (WD) ^-^. - Milk-based formula powder (WF) < (Water with 0.3 ppm F). - Soy-based formula powder. (WD) ^-^. - Soy-based formula powder (WF) < (Water with 0.3 ppm F). - Milk-based formula powder (WD) ^-^. - Soy-based formula powder (WD) ^-^. - Ready-to-Feed, infant milk - Ready-to-Feed, Soy-based - Liquid Concentrate formula. - Liquid Concentrate, soy-based formula.	7 49	0.06 ppm (NR) ^•^ 0.43 ppm (NR) ^•^ 0.34 ppm (NR) ^•^ 0.51 ppm (NR) ^•^ 0.12 ppm (0.08) 0.16 ppm (0.09) 0.15 ppm (0.06) 0.21 ppm (0.10) 0.27 ppm (0.18) 0.21 ppm (0.10)	NR ^•^ (0.05–0.13) NR^•^ (0.40–0.49) NR ^•^ NR ^•^ NR ^•^ NR ^•^ NR ^•^ NR ^•^ NR ^•^ NR ^•^	[60,81]
Australia	- Ready-to-Feed, infant milk - Milk-based formula powder (NRF) ^2^ - Ready-to-Feed, Soy-based - Milk-based formula powder. (Water with 0.0 ppm F). - Milk-based formula powder. (WF) < (Water with 0.2 ppm F). - Milk-based formula powder. (WF) < (Water with 0.7 ppm F). - Milk-based formula powder. (WF) < (Water with 1 ppm F) - Soy-based formula powder. (WF) < (Water with 0.0 ppm F) - Soy-based formula powder. (WF) < (Water with 0.2 ppm F) - Soy-based formula powder. (WF) < (Water with 0.7 ppm F) - Soy-based formula powder. (WF) < (Water with 1 ppm F)	51 33	0.03 ppm (0.010) 0.27 ppm (0.53) 0.56 ppm (0.40) 0.01 ppm (NR) ^•^ 0.04 ppm (NR) ^•^ 0.12 ppm (NR) ^•^ 0.16 ppm (NR) ^•^ 0.02 ppm (NR) ^•^ 0.05 ppm (NR) ^•^ 0.13 ppm (NR) ^•^ 0.17 ppm (NR) ^•^	NR ^•^ (0.00–0.03) NR ^•^ (0.01–1.2) NR ^•^ (0.05–0.91) 0.09 (0.00–0.02) 0.03 (0.04–0.05) 0.11 (0.11–0.13) 0.15 (0.16–0.17) 0.02 (0.01–0.03) 0.05 (0.04–0.06) 0.12 (0.12–0.14) 0.171 (0.16–0.18)	[59]
Thailand	- UHT soy milk - Soy milks	144	0.27 ppm (0.510) 0.24 ppm (0.46)	0.14 (0.0–3.49) 0.14 (0.0–3.49)	[68]
South Korea	- Milk-based formula powder. (WF) < (Water with 0 ppm F) - Milk-based formula powder. (WF) < (Water with 0.5 ppm F) - Milk-based formula powder. (WF) < (Water with 0.8 ppm F) - Milk-based formula powder. (WF) < (Water with 1 ppm F)	20	0.09 ppm (NR) ^•^ 0.50 ppm (NR) ^•^ 0.75 ppm (NR) ^•^ 0.92 ppm (NR) ^•^	NR ^•^ (0.03–0.27) NR ^•^ (0.42–0.71) NR ^•^ (0.65–0.97) NR ^•^ (0.81–1.14)	[62]
New Zealand	- Milk-based formula powder. (WD) ^-^	32	0.069 ppm (NR) ^•^	NR ^•^ (0.02–0.20)	[69]
Poland	- Milk-based formula powder (DW) ^+^ - Milk-based formula powder. (WF) < (Water with 0.5 ppm F) - Milk-based formula powder. (WF) < (Water with 0.8 ppm F) - Milk-based formula powder. (WF) < (Water with 0.9 ppm F) - Milk-based formula powder. (WF) < (Water with 1 ppm F)	27	0.03 ppm (NR) ^•^ 0.35 ppm (NR) ^•^ 0.54 ppm (NR) ^•^ 0.60 ppm (NR) ^•^ 0.66 ppm (NR) ^•^	NR ^•^ (0.01–0.05) NR ^•^ (0.30–0.40) NR ^•^ (0.47–0.61) NR ^•^ (0.52–0.68) NR ^•^ (0.58–0.75)	[61]
Iran	- Milk-based formula powder. (NRF) ^2^	12	1.73 ppm (0.3)	NR ^•^	[41]

^+^ DW: Distilled water. * NFW: Fluoride-free water. ° BW: Bottled water. WF: Fluoride water. ^-^ WD: Deionized water. ^1^ TW: Tap water. ^•^ NR: No report. ^2^ NRF: The form of reconstitution is not reported.

## 5. Conclusions

Fluoride is beneficial in preventing cavities; excessive consumption during tooth development can cause dental fluorosis. This article emphasized the importance of maintaining a delicate balance in fluoride intake, especially in early childhood, to reap the benefits of fluoride and avoid potential adverse effects. We reviewed several studies from different regions of the world that examined fluoride concentrations in milk-based infant formulas. These studies revealed variations in fluoride levels, which can be influenced by factors such as the water source used for reconstitution. Importantly, this research highlights that the fluoride content in water, as well as other sources, can significantly affect a child’s fluoride intake, potentially elevating the risk of dental fluorosis.

## Data Availability

Not applicable.
